# Nanoporous Titanium Surfaces for Sustained Elution of Proteins and Antibiotics

**DOI:** 10.1371/journal.pone.0092080

**Published:** 2014-03-14

**Authors:** Amirhossein Ketabchi, Kristopher Komm, Malaika Miles-Rossouw, Davide A. D. Cassani, Fabio Variola

**Affiliations:** 1 Department of Mechanical Engineering, University of Ottawa, Ottawa, Canada; 2 Department of Chemical and Biological Engineering, University of Ottawa, Ottawa, Canada; 3 Department of Chemistry, Material and Chemical Engineering, Politecnico di Milano, Milan, Italy; 4 Department of Physics, University of Ottawa, Ottawa, Canada; Russian Academy of Sciences, Institute for Biological Instrumentation, Russian Federation

## Abstract

Current medically relevant metals for prosthetic reconstructions enjoy a relatively good success rate, but their performance drops significantly in patients with compromised health status, and post-surgical infections still remain an important challenge. To address these problems, different nanotechnology-based strategies have been exploited to create implantable metals with an enhanced bioactivity and antibacterial capacities. Among these, oxidative nanopatterning has emerged as a very effective approach to engender nanoporous surfaces that stimulate and guide the activity of adhering cells. The resulting nanoporosity is also attractive because it offers nanoconfined volumes that can be exploited to load bioactive compounds and modulate their release over time. Such extended elution is needed since a single exposure to growth factors and/or antibiotics, for instance, may not be adequate to further sustain bone regeneration and/or to counteract bacterial colonization. In this article, we assessed the capacities of nanoporous titanium surfaces generated by oxidative nanopatterning to provide controlled and sustained elution of proteins and antibiotic molecules. To this end, we have selected bovine serum albumin (BSA) and vancomycin to reflect commonly used compounds, and investigated their adsorption and elution by Fourier-transform infrared (FT-IR) and ultraviolet–visible (UV-VIS) spectroscopy. Our results demonstrate that while the elution of albumin is not significantly affected by the nanoporosity, in the case of vancomycin, nanoporous surfaces provided an extended release. These findings were successively correlated to the establishment of interactions with the surface and physical-entrapment effects exerted by the nanopores, ultimately highlighting their synergistic contribution to the release profiles and thus their importance in the design of nanostructured eluting platforms for applications in medicine.

## Introduction

Nanostructured materials that effectively promote biological events promise to play a major role in advancing current biomedical implants.[1], [2] In addition to enhanced bioactive properties, the next generation of implantable materials should also feature the capacity to provide sustained elution of growth factors and/or antibiotics to further support bone regeneration and/or counteract bacterial colonization.[3] For these reasons, the development of new materials capable of simultaneously serving multiple purposes (i.e. bioactivity coupled with drug-elution properties) has become an important prerequisite for better-performing biomedical devices.[4] To this end, significant efforts have been made to engender bioactive hollow nanostructures on implantable metals (particularly titanium, the gold standard in implantology).[1], [5] The resulting porous nanotopographies are in fact expected to simultaneously exert direct cueing to the surrounding biological environment while providing nanoconfined volumes that can be advantageously exploited to load bioactive agents and modulate their release over time.

In this context, titania nanotubular surfaces created by anodization have shown an enhanced bioactivity conjugated with the capacity to store diverse compounds and control their elution.[6]–[9] In parallel with these studies, oxidative nanopatterning has emerged as an effective alternative to improve the biological response to titanium, as a result of the creation of nanoporous surfaces capable of direct physicochemical cueing to cells.[10]–[13] Compared to the significantly more voluminous titania nanotubes (diameter and length range from tens to hundreds nanometers),[7], [14] nanoporous surfaces engendered by oxidative nanopatterning are characterized by a 40–50 nm-deep 3-dimensional network of smaller pores (20–25 nm in diameter).[15], [16] While the bioactive properties of such surfaces have been already highlighted and characterized,[10]–[13] their potential to act as nanoengineered platforms for sustained elution of bioactive agents has however never been studied.

To address this challenge, we investigated the elution properties of the nanoporosity without the use of intermediate linking agents, a strategy that, although effective,[17] may alter, and even offset, the contribution of underlying nanostructures.[18] To this end, we have chosen a protein (i.e. bovine serum albumin, hereafter indicated as BSA) and an antibiotic (i.e. vancomycin hydrochloride, hereafter indicated as vancomycin). BSA is a heart-shaped protein with dimensions of 84×84×84×31.5 Å.[19], [20] Vancomycin, 32×22 Å in size, is one of the most effective antibiotic molecules against gram-positive bacteria.[8], [21]–[23] Such a choice was motivated by the following factors: (*i*) the size of both agents theoretically allows them to fit in the nanopores; (*ii*) although BSA does not posses any therapeutic application, its common use as a model protein (to exemplify the delivery of growth factors, for instance) relates our work to previous literature;[8], [22] (*iii*) vancomycin opens the door for the potential use of nanoporous surfaces in antibacterial applications in medicine.

In our study, we initially loaded BSA and vancomycin onto smooth and nanoporous surfaces, thereby creating four different experimental groups. Distinctively from previous work,[6]–[9], [17], [22] we first focused on understanding how the initial interactions with the surface may affect the resulting elution by exploiting Fourier-transform infrared (FT-IR) spectroscopy. We then exploited ultraviolet–visible (UV-VIS) spectroscopy to quantify release profiles and elution rates, ultimately relating them to the FT-IR findings. Our results demonstrate that, similarly to titania nanotubes, nanoporous surfaces synergistically combine bioactivity with drug-delivery capabilities, and thus offer promising capacities for various applications in medicine.

## Materials and Methods

### Preparation of nanoporous surfaces (oxidative nanopatterning)

Grade 2 titanium disks, 12 mm in diameter and 1 mm in thickness, were mechanically polished with a 2-step process by the supplier (Firmetal Co., Ltd., Shanghai, China). Before treatment, as-received disks were first cleaned in toluene in an ultrasonic bath for 20 min and successively rinsed in deionized water and ethanol. A 50:50 mixture of H_2_SO_4_ (37N, Fisher Scientific)/H_2_O_2_ (30%, Fisher Scientific) was used to generate nanoporous surfaces. Titanium disks were immersed in freshly prepared etching solutions at room temperature for 2 hours according to a previously established protocol.[16]

### Loading of BSA and vancomycin

300 μL of solutions containing 50 mg/mL of bovine serum albumin (Sigma-Aldrich) and vancomycin hydrochloride (Sigma-Aldrich) in 10% ethanol were pipetted onto smooth and nanoporous titanium disks. Ethanol was used to facilitate the penetration of the solution into the 3-dimensional network of nanopores by reducing the surface tension of the water. In fact, it has been demonstrated that ethanol favours the diffusion of molecules into nanoporous matrices by increasing surface wettability and capillary driving force.[24], [25] In this context, ethanol is known to influence the solubility of BSA as well as its secondary structure. However, for concentrations below 30%, the effects on the protein's solubility and its denaturation are minor.[26] BSA- and vancomycin-loaded disks were successively dried overnight in a vacuum oven at room temperature. After drying, the excesses of the loaded agents were removed by scraping with a plastic blade and thorough rinsing with deionized water to minimize the amount of molecules not directly adsorbed onto surfaces. Because of the experimental challenges in consistently collecting the amounts of BSA/vancomycin removed with these last two steps, we could not precisely determine the initial loaded mass (see Results).

### Scanning electron microscopy (SEM)

To confirm the presence of the characteristic network of nanosized pores,[10], [16] four randomly selected treated samples were imaged with a field emission scanning electron microscope (JEOL-JSM7400F SEM) operated at 1.5 kV. Polished controls were also imaged for comparison.

### Atomic force microscopy (AFM)

Variations in the surface micro and nanotopography were characterized by AFM and quantified in terms of root-mean-square (RMS) roughness. To this end, a WITec Alpha 300R confocal Raman microscope (WITec, Ulm, Germany) in non-contact mode with a triangular silicon tip was used. Images were successively processed with the WSxM software (Nanotec Electronica, Madrid, Spain) to calculate RMS values of four randomly selected 4×4 μm and 500×500 nm areas.

### Fourier-transform infrared spectroscopy (FT-IR)

A Nexus 870 FT-IR spectrometer equipped with the SAGA accessory (smart aperture grazing angle, 80° with respect to the surface normal; Thermo Nicolet) with an 8-mm-diameter opening was used to investigate BSA- and vancomycin-loaded samples before elution. For each of the four experimental groups, four (vancomycin) and five (BSA) samples were analyzed (i.e. for vancomycin-control and vancomycin-nanoporous: *n* = 4; for BSA-control and BSA-nanoporous: *n* = 5).

Spectroscopic information was collected in the 400–2000 cm^−1^ range with a 4 cm^−1^ resolution (256 scans per spectrum), using a gold substrate for reference. Such choice allowed us to (*i*) capitalize on previous literature to interpret FT-IR data and investigate the adsorption of BSA and vancomycin onto substrates (in the 1200–1800 cm^−1^ range),[27]–[32] and (*ii*) monitor the band in the 500–1100 cm^−1^ region and provide one additional non-destructive quality control step to further ensure the creation of the nanoporous layer in treated samples (data not shown. The reader can refer to references[11], [16], [33]).

Infrared data were analyzed by using the OriginPro software (OriginLab corporation, Nothampton, MA). Spectra were smoothed with the adjacent-averaging method and, after linear baseline subtraction, fitted with Gaussian functions to resolve secondary vibrational components according to previously published literature.[27]–[32] The same software was also used to carry out the independent two-sample *t*-test to assess statistically significant differences (P<0.05).

### UV-VIS spectroscopy

BSA- and vancomycin-loaded disks were immersed in custom-made cylindrical holders containing deionized water and placed on a horizontal shaker at 37 °C. The holder was sealed to prevent evaporation. For each of the four experimental groups, all elution experiments were carried out at least in quadruplicate. Constant-volume aliquots were collected at various time points and analyzed by exploiting an UV-VIS spectrophotometer (Biotek Epoch, Fisher Scientific). Equal amounts of deionized water were added every time the samples were taken to maintain a constant volume. This approach also allowed us to mimic the *in vivo* renal extraction of vancomycin, as previously described.[18] In the case of BSA, a Bradford protein assay (Sigma-Aldrich) was used. Absorbance was measured at a wavelength of 595 nm and 280 nm for BSA and vancomycin, respectively.[18], [34] The mass eluted at every time point was calculated based on the calibration curves obtained with known concentrations of the two experimental agents in deionized water, and adjusted to account for variations in concentration due to the systematic removal and replacement of aliquots. In particular, the eluted mass in 150 μL aliquots (measured by UV-VIS) taken at the time interval t_i_ was calculated by accounting for the mass removed from the system by the aliquot taken at the interval t_i-1_ (which was replaced with an equal amount of deionized water). The mass eluted at every interval (M_t_) was then normalized to the mass eluted at one week (M_168h_) under the assumption that the release was complete at this point.

For each sample, we then calculated the elution rate *r* (i.e. mass eluted divided by the time interval) at different time points (*r*_t_) and normalized it to the rate of the initial burst (*r*_burst_, t = 15 minutes). We successively averaged the resulting relative values and plotted them in a graph that displays the elution trend over the logarithm of time.

### Data fitting

The fraction of mass eluted (i.e. M_t_/M_168h_) was analyzed with the following semi-empirical equation:[35]

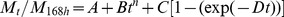
(1)


Such model assumes that three processes are implicated, namely:[35] (*i*) initial burst release; (*ii*) diffusion-controlled elution[36], [37]; (*iii*) dissolution with a faster asymptotic profile. Firstly, because at t = 0 there is theoretically no elution, we have imposed the condition M_0_/M_168_ = 0 (which yielded A = 0). Secondly, for each elution curve we computed the diffusion exponential *n* by fitting the experimental data for which M_t_/M_168_≤0.65 with the second term of the equation (Bt^n^). This allowed us to obtain the best compromise between the validity of the model[36]–[39] and the reliability of the analytical fitting procedure. For each curve, we successively imposed the corresponding diffusion exponential in equation (1) to determine the parameter D.

For the analysis of the experimental relative elution rates (i.e. *r*_t_ */r*_burst_), the following exponential decay function was used:
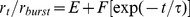
(2)


The parameter τ (decay constant), calculated within the first 8 hours of elution (interval which exhibits the most different behaviors), was used to compare elution rates.

## Results

**Figure 1:**
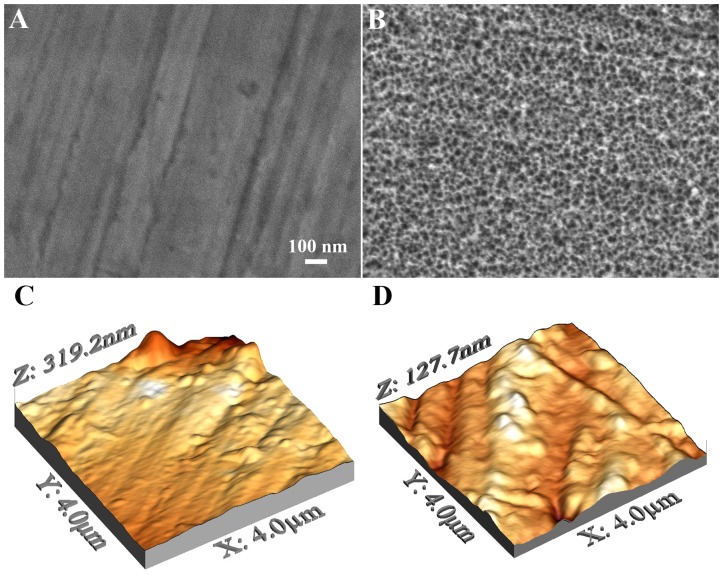
SEM micrograph of controls (A) and nanoporous surfaces (B) generated on titanium by oxidative nanopatterning. While controls are smooth at the nanoscale (micrometric marks due to the mechanical polishing were visible), treated surfaces display the characteristic sponge-like nanostructure. AFM 4×4 μm micrographies of controls (C) and treated (D) titanium.

In agreement with previous work,[10], [11], [15], [16] while polished controls did not show any reproducible feature at the nanoscale,[16] treated surfaces exhibited the characteristic 3-dimensional network of nanometric pits. Figures 1A and B display SEM images of titanium surfaces before and after oxidative nanopatterning. Untreated disks exhibited a smooth surface at the nanoscale with no distinctive topographical features (Figure 1A). Only marks due to the mechanical polishing were observed. Conversely, a reproducible 3-dimensional sponge-like porosity characterized by nanosized pores (about 20 nm in diameter) uniformly distributed across the surface was observed on treated samples (Figure 1B). While the majority of the nanopores appeared isolated with well-defined edges, bigger pores resulting from the interconnection of single pits were also observed. Such size distribution has been previously characterized in the case of nanoporous titanium and Ti6Al4V alloy.[11], [16]

AFM analysis determined that the RMS roughness values calculated within the micrometric areas were 47±12 and 68±13 nm for control and nanoporous samples respectively, showing minimal differences in the surface microtopographies. Consistently with our previous results,[16] RMS roughness values measured in the nanometric areas were 8±4 nm and 17±5 nm for controls and treated samples, respectively.

**Figure 2:**
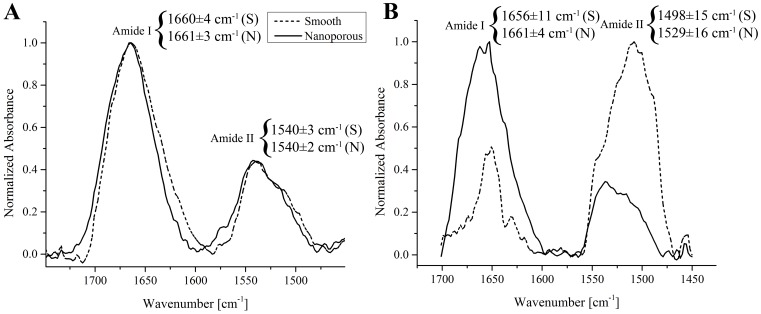
Representative examples of the FT-IR spectrum of bovine serum albumin (A) and vancomycin (B) adsorbed onto smooth (dashed line) and nanoporous (solid line) surfaces. The amide I and II maxima are indicated (S: smooth; N: nanoporous).

Figure 2A shows representative examples of FT-IR spectra of BSA adsorbed onto smooth and nanoporous surfaces before elution. Consistently with previous literature, two bands appeared in the 1600–1700 and 1480–1600 cm^−1^ regions. These were assigned to the amide I and II, and correspond to the C = O stretching (υ_C = O_) and N-H bending (δ_N-H_, i.e. vibrations of C-N-H angle) vibrations, respectively.[27], [30], [40], [41] The amide I/II ratio, as well as the position of the maxima of the amide I and II bands, were used to monitor conformational changes on BSA after the adsorption process.[27], [42] Qualitative analysis of the FT-IR spectra of BSA did not reveal any significant difference in the absorption bands. In fact, the amide I and II bands of the protein adsorbed onto controls and nanoporous surfaces overlapped notably, thereby suggesting no major conformational changes induced by the substrate. Qualitative analysis was corroborated by peak deconvolution and fitting, which allowed us to investigate specific aspects of BSA adsorption.[16], [27], [28] In particular, the amide I/II ratio, calculated at the maxima of the two bands, showed similar values on smooth and nanoporous surfaces (i.e. 1.9±0.8 and 2.2±0.4). In addition, the maxima of the amide I and II bands, positioned at 1660±4 cm^−1^/1540±3 cm^−1^ (smooth controls) and 1661±3 cm^−1^/1540±2 cm^−1^ (nanoporous surfaces),[30] did not undergo significant shift induced by the substrate.

Conversely, Figure 2B reveals clear differences in the FT-IR spectra of vancomycin adsorbed onto smooth and nanoporous surfaces before elution. Similarly to BSA, the two bands in the 1600–1700 and 1485–1570 cm^−1^ regions were assigned to amide I and II, respectively.[27], [29]–[32], [40], [41] It can be observed from the representative examples shown in Figure 2B that the relative intensity of the amide I band increased and that of the amide II band decreased from smooth to nanoporous surfaces. Concomitantly, the amide II band shifted towards higher wavenumbers. The relative intensity variations observed were successively quantified in terms of amide I/II ratio calculated at the maxima of the two bands, and resulted to be 0.5±0.3 and 1.9±0.9 for smooth and nanoporous surfaces, respectively. In addition, both bands shifted in relation to the substrate, and this was more pronounced for the amide II. More precisely, the maxima of the amide I band were located at 1656±11 cm^−1^ and at 1661±4 cm^−1^, and those of the amide II band at 1498±15 cm^−1^ and at 1529±16 cm^−1^ on smooth and nanoporous surfaces, respectively.

**Figure 3:**
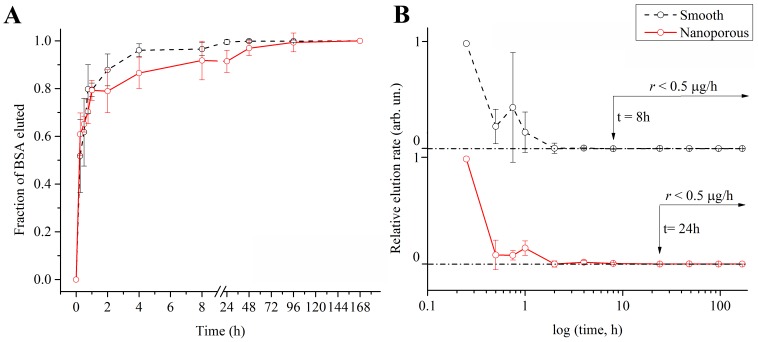
Point-by-point curves of the fraction eluted (A) and relative elution rate (B) of bovine serum albumin adsorbed onto smooth (solid black line, *n* = 4) and nanoporous (solid red line, *n* = 6) surfaces. Dotted line in B represents the plateau of no significant elution (i.e. elution rate<0.5 μg/h). Data points are visualized as mean values with standard deviation.

**Figure 4:**
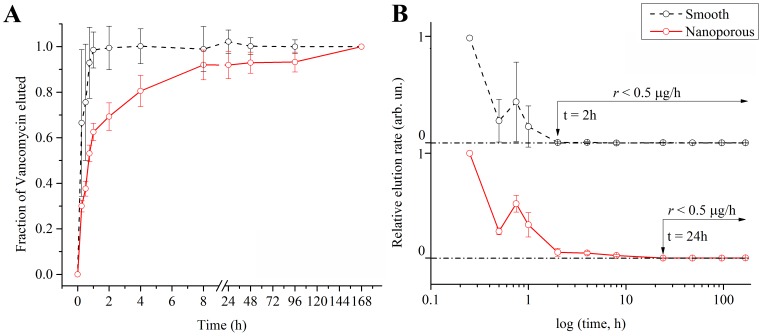
Point-by-point curves of the fraction eluted (A) and relative elution rate (B) of vancomycin adsorbed onto smooth (solid black line, *n* = 4) and nanoporous (solid red line, *n* = 4) surfaces. Dotted line in B represents the plateau of no significant elution (i.e. elution rate<0.5 μg/h). Data points are visualized as mean +/− standard deviation.

During the elution experiments, we monitored the release of BSA (Figure 3A) and vancomycin (Figure 4A) at various intervals, from 15 minutes to one week (168 hours). This approach simultaneously allowed us to (*i*) operate within vancomycin *in vitro*'s half-life (i.e. approximately 9 days)[18] and (*ii*) link our investigation to potential *in vivo* antibacterial applications (see Discussion).

In the case of BSA, compared to smooth controls, nanoporous surfaces did not significantly impact the protein release. In fact, the two curves overlapped at the beginning (i.e. 15 minutes–2 hours) and at the end (i.e. 48 hours onwards) of the elution, slightly diverging at 4 and 24 hours (P<0.05) (Figure 3A). In order to further our investigation to clearly visualize and compare release trends, we plotted the normalized elution rates (Figure 3B). In general, the elution provided by both smooth and nanoporous decreased significantly after the first 15 minutes, and achieved a plateau (dotted line in Figure 3B) approximately at the same time. To better compare the two behaviors, we chose from the absolute data sets an arbitrary rate (i.e. 0.5 μg/h) within the plateau region and assigned it to the termination of significant elution. We then linked this reference value to its corresponding relative rate, and visualized it in Figure 3B. In this way, we could better determine that smooth surfaces reached a steady state slightly before nanoporous ones (8 hours vs 24 hours).

A similar approach was adopted to investigate the release profiles of vancomycin (Figure 4A). As anticipated by the FT-IR results, the behavior of the antibiotic molecules was affected by the substrate. In particular, Figure 4A shows a clear difference in the elution profiles of nanoporous surfaces (P<0.05 for the whole interval, except at t = 15 min and 8 hours), which provided a slower and more sustained release. In fact, while smooth surfaces completed the elution within the first hour, the release from nanoporous ones was not concluded even after 96 hours (i.e. 93±4% of the total elution at one week). Such behavior was also observed by plotting the normalized elution rates (Figure 4B), which clearly showed a delay of nanoporous surfaces in reaching the plateau. This was quantified by comparing the time required to arrive at the set arbitrary value of 0.5 μg/h, and resulted to be 2 hours for smooth and 24 hours for nanoporous surfaces.

From elution data we could also estimate the initial loaded mass by determining the total mass eluted after 168 hours, under the assumption that the elution was complete at this point. Although these values may underestimate the actual loaded mass, they showed that treated samples retained higher amount of BSA/vancomycin throughout the loading phase (i.e. BSA: smooth = 17±6 μg/nanoporous = 97±51 μg; vancomycin: smooth = 45±43 μg/nanoporous = 106±60 μg).

**Figure 5:**
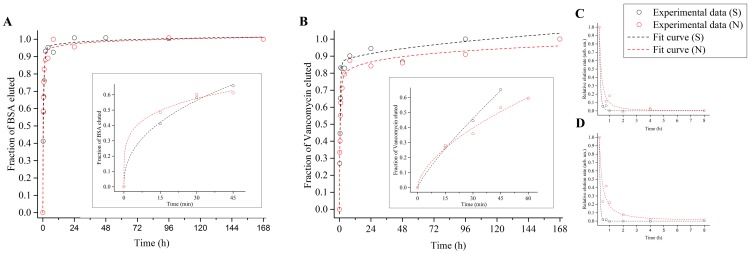
Representative examples of the fitting process. (A) Elution of BSA fitted with equation (1) and calculation of the elution exponential *n* (inset); (B) elution of vancomycin fitted with equation (1) and calculation of the elution exponential *n* (inset). (C) Relative elution rate of BSA fitted with equation (2); (D) relative elution rate of vancomycin fitted with equation (2).

Figures 5 A–D show the results obtained by fitting experimental data with equations (1) and (2). The parameters *n*, D and τ were calculated independently for each elution curve, and the resulting values were then averaged for all the samples tested (Table 1). In the case of BSA, *n* was lower than 0.5 on both surfaces, and no significant differences were detected by the *t*-test. Similarly, the diffusion exponential showed no statistical differences for vancomycin, displaying however an average value of 0.5 on both substrates. The fitting procedure also revealed that, while for BSA the coefficient D did not vary statistically, it was higher for vancomycin eluted from smooth surfaces. In addition, while the decay coefficients were identical for the protein, in the case of the antibiotic they were higher for the elution from nanoporous surfaces.

**Table 1:** Selected fitting parameters for equations (1) and (2).

	BSA (S)	BSA (N)	P-value	Vancomycin (S)	Vancomycin (N)	P-value
*n*	0.3±0.1	0.2±0.02	>0.05	0.5±0.2	0.5±0.05	>0.05
D	2.2±0.7	3.1±0.3	>0.05	1.9±0.3	1.3±0.2	<0.05
τ	0.2±0.1	0.2±0.07	>0.05	0.4±0.1	0.6±0.08	<0.05

Values are expressed as mean +/− standard deviation. A P-value <0.05 indicates statistically significant differences determined by the independent two-sample *t*-test.

## Discussion

In this article, we assessed the elution capacities of a 3-dimensional network of nanosized pores created on titanium by oxidative nanopatterning (Figure 1). Previous physicochemical characterization (detailed in references[10], [16]) established that the resulting surfaces are characterized by a hydrophilic and contaminant-free, 3-dimensional array of nanometric pits ranging between 20 and 25 nm in diameter and extending for 40–50 nm in depth. Such chemically generated nanoporosity is also characterized by a greater RMS roughness at the nanoscale, as compared to controls.

It appeared evident from the early stages of our study that the investigation of how BSA and vancomycin interact with smooth and nanoporous surfaces after the loading phase was a fundamental prerequisite to understand the resulting elution profiles. Our working hypothesis asserted in fact that the establishment of specific interactions with the surface and, in the case of nanoporous substrates, the potential physical entrapment exerted by nanoconfined volumes, would impact the tendency of the molecules to desorb, ultimately dictating the overall release profile.[42] To validate our assumptions, we exploited infrared spectroscopy, a powerful tool to characterize the structure, stability, conformational modifications and reaction mechanisms of proteins and complex molecules in a wide variety of environments.[41], [43] In particular, we employed Fourier transform infrared spectroscopy (FT-IR), a technique that has been successfully used to investigate the adsorption of BSA onto various substrates[28], [42], [44], [45] as well as structural changes and interactions of vancomycin [40], [46].

In this context, the loading protocol we adopted was characterized by experimental variability from sample to sample. In particular, the scraping and rinsing steps to remove the excess of adsorbed proteins and antibiotic molecules was not perfectly consistent across different samples, and this translated into variations in the total loaded mass. Such differences affected the FT-IR band intensities, thereby making precise spectroscopic quantification and comparison unreliable. In addition, quantitative inaccuracy could also originate from the different optical paths of the IR beam between control and treated samples.[33] These limitations were however overcome by considering the relative amide I/II ratio. The absolute peak shifts within the amide regions were nonetheless examined since these were not expected to depend on the amount of adsorbed mass.

FT-IR analysis showed no evident differences in the absorption bands of the protein adsorbed onto smooth and nanoporous surfaces, which displayed the characteristic features of BSA adsorbed on solid substrates[27], [28] (Figure 2A). The lack of variations in both the amide I/II ratio and band maxima positions indicates that the nanometric pores do not significantly affect the protein structure, as compared to smooth surfaces. However, although undetected by FT-IR, given its relatively larger size and more complex structure, we cannot completely exclude less evident effects exerted by the surface on BSA conformation.

In the case of vancomycin, clear differences were detected in the FT-IR spectra in relation to the substrate (Figure 2B). Such variations in the IR bands were previously ascribed to interactions between vancomycin's NH and CO_2_ groups.[46] In particular, the inversion of relative intensities and significant blue shift of the amide II band were associated to the creation of H-bonding interactions between carboxylate and amide groups.[46], [47] We can thus extrapolate that the observed FT-IR results stemmed from different interactions between vancomycin's side groups, and that these were affected by the substrate.[29] Noteworthy, FT-IR spectra on nanoporous surfaces were consistent with those of the pristine antibiotic.[29], [31] This, while provides evidence that no modifications that can potentially alter vancomycin's bacterial efficiency occurred, also indicates that smooth controls most likely interfered with the formation of H-bonding interactions. These differential effects can be explained by the fact that smooth and nanoporous surfaces not only show different nanotopographies, but also differ in other physicochemical parameters that can ultimately influence the interactions with molecules (eg surface chemistry, presence of contaminants and wettability).[15], [16] In addition, compared to BSA, the larger variations of the positions of the band maxima likely depended on alterations of secondary bands within the amide I and II regions, and this occurred on both substrates. The systematic analysis of such spectral rearrangements lies beyond the scope of the present study. However, since these phenomena have been associated to the effects of binding processes during complexation of vancomycin,[29] this further provides evidence of substrate-dependent molecular interactions.

Elution profiles and release rates (Figures 3 and 4) were obtained by (*i*) normalizing the calculated eluted mass at each time point to that eluted at one week and (*ii*) normalizing the calculated elution rate to the initial burst release rate, respectively. This approach permitted us to dissociate the elution profiles from the initial mass adsorbed on the disks, thereby allowing us to solely investigate the kinetics of the release while overcoming the experimental variability in the loaded mass.

In the case of BSA, because the two elution curves diverged in two points (Figure 3A) and the release from nanoporous surfaces reached a steady state later than from smooth ones (Figure 4B), we can speculate that the nanoporosity is still capable of exerting influence on this protein. The effects, however, were too feeble to significantly affect the resulting overall elution profile.[12]

The contextualization and comparison of our findings with BSA in relation to the performance of similar surfaces, namely titania nanotubes, were not univocal, since published results varied according to the protein concentration as well as on the nanotube height and diameter. For instance, the elution interval values can range from 65 minutes (800 μg of BSA in 80×400 nm nanotubes) to 30 days (100 μg of BSA in 100 nm×5 μm nanotubes).[8], [22] In addition, a direct comparison between smooth controls and nanotubular surfaces in relative terms (i.e. fraction of BSA eluted), fundamental to compare diverse elution profiles independently on the total loaded mass (likely greater on nanotubular surfaces because of the greater volume offered by the hollow structures),[8] was not found. Based on our results and our knowledge of previous literature, we can only conclude that nanoporous surfaces completed the elution of BSA over an interval which lies within the timeframe reported for nanotubular surfaces. The specific contribution of the nanoporosity to such extended release was however minimal.

Conversely, as revealed by the FT-IR analysis (Figure 2B), vancomycin interacted differently with smooth and nanoporous surfaces. Such a differential behavior may have contributed to affecting the release profiles. In the design of the elution experiments, in particular in the selection of the time point distribution as well as of the maximum interval, we carefully kept into account vancomycin's *in vitro* half-life[18] and its potential applications in tissue engineering. More precisely, we concentrated the time points within the first 8 hours, and extended our investigation to a maximum of one week in order to fully cover the interval required for infection to develop. In fact, bacterial attachment and adhesion require a matter of hours to develop, while aggregation and dispersion can occur as early as few days after attachment.[48] In addition, we also considered that vancomycin has a 4–6 hours of *in vivo* life before being removed by renal extraction.[18], [49] Therefore, a sustained elution of vancomycin to contrast the onset and development of bacterial infection may only be needed in the short term. Nanoporous surfaces showed the capacity to provide a slower elution within such an interval.

During the *in vitro* experiments, the maximum concentration of vancomycin at 168 hours calculated by UV-VIS never exceeded 500 μg/mL. As previously indicated,[18] the optimal therapeutic concentration of vancomycin to efficiently contrast *S. aureus* and *S. epidermidis* is 16 μg/mL, and should never be greater than 80 μg/mL in order to avoid ototoxic effects. However, since the acceptable local concentration near the implant's surface is expected to be higher than the systemic one,[18] it is difficult to estimate whether the maximum values measured in our experiments, significantly greater than the *in vivo* ototoxicity threshold, are still within an adequate range for clinical applications. It follows that a careful validation of the precise *in vivo* requirements for a specific therapeutic application is a fundamental step in rational design of drug-eluting platforms capable of providing the optimal concentrations.

The analytical computation of the diffusion exponent *n* showed that the elution mechanism was not statistically influenced by the substrate. However, while for BSA the elution mechanism resulted to be a quasi-Fickian diffusion (*n*<0.5)[36], [38], [50], data fitting indicated a Fickian diffusion for vancomycin (*n* = 0.5, as previously reported for antibiotics eluting from nanoporous templates[51]).[35], [36], [38], [50] In this context, we would like to point out that the variation of *n* for vancomycin eluted from smooth controls does not allow to precisely determine the elution mechanism. This most likely resulted from the combination of various factors (e.g. the loading protocol and the intrinsic properties of control surfaces) which prevented the consistent adsorption and retention/elution of molecules. However, in the case of nanoporous surfaces, the assessment of the mechanism was reliable as a result of a greater consistency in the experimental data. The computed values for D and τ did not indicate significant differences with BSA, validating experimental data. Conversely, they confirmed a slower elution from nanoporous surfaces in the case of vancomycin. These two parameters express in fact how fast models (1) and (2) approach the horizontal plateaus in Figures 3–4 B–C.

For both experimental agents tested in this study, the effects exerted by the substrates on their behaviors were first studied by FT-IR analysis, and associated to substrate-dependent interactions (or lack thereof). Our initial expectation that the interactions of any molecule with the nanoporous surfaces were going to be synergistically enhanced by the 3-dimensional nanoporosity as a result of (*i*) the physical entrapment exerted by nanoconfined volumes, (*ii*) the increased surface area and (*iii*) the creation of additional binding sites,[16] was only confirmed with vancomycin. In the case of BSA, in fact, this was not verified. In particular, although nanoporous surfaces showed a higher mass retention throughout the scraping/rinsing steps (likely associated to the physical entrapment), their contribution during elution was marginal. In other words, the nanopores did not provide any significant advantage to prevent the protein from diffusing when loaded samples were immersed in deionized water. This finding can be related to previous work which employed the Quartz Crystal Microbalance (QCM-D) to show that the adsorption efficiency of various proteins/molecules on nanoporous surfaces depended on their intrinsic properties.[52] In particular, it was shown that the adsorption of BSA was limited. Taken together, we can infer that the factors that caused such a limitation (e.g. protein conformation, size, isoelectric point, etc.) could also be responsible for the absence of significant effects on its elution.

In the case of vancomycin, spectral rearrangements that suggest substrate-dependent interactions occurred on both smooth and nanoporous surfaces, but these were more dominant on the former. However, since relatively larger variations in the position of the amide II band were nonetheless detected on treated samples, we cannot completely exclude the presence of nanoporosity-induced substrate-molecule and molecule-molecule interactions. Therefore, while it is apparent that oxidative nanopatterning endows titanium with the capacity of a slower elution of vancomycin, we cannot unequivocally determine whether this solely depends on the nanoporosity, on the establishment of specific interactions, or a synergistic combination of the two.

In conclusion, while the morphological (e.g. total open volume of interconnected porosity) and geometrical (e.g. pore diameter) characteristics of nanoporous surfaces are undoubtedly important for loading significant amounts of bioactive agents and ensuring the required therapeutic concentrations, they may not necessarily be the only parameters that govern the adsorption/elution of proteins/molecules and dictate the release profile. At constant temperature, the efficiency and characteristics of the elution more likely depend (*i*) on synergistic effects exerted by its 3-dimensional physicochemical environment and (*ii*) on the intrinsic properties of the selected bioactive agent. Therefore, in the design of platforms for controlled elution, morphological features as well as the interactions with the surface are all equally important factors to consider. In other words, for a particular nanoporosity, the rational selection of bioactive agents in relation to their affinity to the surface should be addressed in order to create more efficient and better performing eluting nanoporous platforms.

## Conclusions

In this article, we reported the eluting capacities of nanoporous surfaces generated on titanium by oxidative nanopatterning. Our results show that, despite the open volume and pore diameter offered by the 3-dimensional porosity are significantly smaller than that provided by similar hollow nanostructures, nanoporous substrates can nonetheless provide extended elution. Our results indicated that the elution of a model protein, bovine serum albumin, was not significantly impacted by the nanoporosity. This ultimately highlighted and reinforced the importance of the interactions/affinity between bioactive agents and the surface in the design of drug-eluting platforms. Conversely, in the case of vancomycin, we have demonstrated that nanoporous surfaces possess promising abilities for antibacterial applications in medicine. While the approach we adopted has undoubtedly some limitations, its simplicity would permit its direct translation to metallic biomedical implants. To this end, future efforts will focus on optimizing and fine-tuning the experimental protocol to precisely load and deliver the required therapeutic concentrations of selected bioactive agents for *in vivo* applications.[18] The use of intermediate linkers (e.g. silanes)[17] and/or biocompatible coatings (e.g. chitosan),[53] will provide further flexibility in the modulation of elution profiles.
